# 4198 Investigating the Role of Rab27B in Non-Small Cell Lung Cancer Tumor Initiating Cells

**DOI:** 10.1017/cts.2020.306

**Published:** 2020-07-29

**Authors:** Danielle Beetler, Kayleah Beltran, Kayla Lewis, Verline Justilien

**Affiliations:** 1 Mayo Clinic

## Abstract

OBJECTIVES/GOALS: Rab27B, a small GTPase, functions in exosome formation and secretion. Rab27B is overexpressed in non-small cell lung cancer (NSCLC) and predicts patient survival; however, little is known about its importance in NSCLC cells. Here, we investigated the role of Rab27B in NSCLC tumor initiating cells. METHODS/STUDY POPULATION: Tumor initiating cells (TICs) were enriched in a panel of NSCLC cell lines using low adherence spheroid cultures. QPCR and immunoblot analysis were used to compare Rab27B mRNA and protein expression, respectively, in adherent bulk cancer cells and TIC cultures. Lentiviral-packaged short hairpin RNAs (shRNAs) were used to knockdown Rab27B in PC9 and H1299 NSCLC TICs. The effects of Rab27B knockdown on PC9 and H1299 TIC expansion, transformed growth, and invasion were analyzed by MTT cell proliferation, soft agar colony formation, and Boyden chamber assays respectively. RESULTS/ANTICIPATED RESULTS: Quantitative PCR and immunoblot analysis showed that Rab27B expression is elevated in NSCLC TICs when compared to adherent bulk cancer cells. Efficient knockdown of Rab27B was achieved in PC9 and H1299 NSCLC TICs using two independent shRNA constructs. Rab27B knockdown cells exhibited decreased expansion as spheroid cultures, transformed growth, and invasion when compared to non-target shRNA control cells. Future experiments will focus on determining the importance of Rab27B in TIC exosome production and *in vivo* tumor growth and metastasis. DISCUSSION/SIGNIFICANCE OF IMPACT: Our results show that Rab27B is important in NSCLC TIC growth and invasion. Further studies are needed to determine the mechanism of Rab27B action. TICs have been linked to enhanced tumorigenic properties, suggesting that Rab27B could be a good candidate for therapeutic targeting of NSCLC TICs.

